# Estimated carbon emissions for PBS‐subsidised prescription respiratory inhalers, Australia, 2019–2023: a descriptive analysis

**DOI:** 10.5694/mja2.52715

**Published:** 2025-06-26

**Authors:** Luise Kazda, Alexandra L Barratt, Sean Docking, Kristen Pickles, Darlene Cox, Ross S Bailie, Kate Wylie, Katy JL Bell

**Affiliations:** ^1^ Health Research Institute, University of Canberra Canberra ACT; ^2^ Sydney School of Public Health, the University of Sydney Sydney NSW; ^3^ Monash University Melbourne VIC; ^4^ Health Care Consumers' Association Canberra ACT; ^5^ Doctors for the Environment Australia Melbourne VIC

**Keywords:** Asthma, Chronic obstructive pulmonary disease, Respiratory system agents, Epidemiologic measurements, Climate change

The Australian National Health and Climate Strategy^1^ identifies reducing the use of pressurised, metered dose inhalers as a high priority for decarbonising health care. These high emissions inhalers contain potent greenhouse gases as propellants, producing ten to thirty times as much in carbon dioxide equivalent (CO_2_e) emissions as low emissions inhalers (dry powder and soft mist inhalers), which are often clinically equivalent.

To investigate the carbon footprint of inhalers dispensed in Australia and subsidised by the Pharmaceutical Benefits Scheme (PBS), we analysed aggregate‐level PBS dispensing data for the period 1 January 2019 – 30 November 2023, based on United Kingdom estimates of emissions per inhaler.^2^ We aggregated the estimated emissions for all inhalers dispensed by year and type, and summarised age‐standardised population rates of inhaler dispensing. We then conducted time series analyses of monthly values, adjusted for autocorrelation, to derive regression trend lines; we estimated mean monthly percentage changes (MMPCs) using joinpoint regression analysis. For short‐acting β_2_ agonist inhalers, we assumed that a mean 1.5 inhalers were dispensed per script; in a sensitivity analysis, we assumed a mean of one inhaler was dispensed per script (further details: Supporting Information, part 1). The University of Canberra human research ethics committee exempted the study from formal ethics review (HREC 2024/14042). Access to the data and approval for the publication of findings based on the data were granted by Services Australia as data custodian.

The number of PBS‐subsidised inhalers dispensed increased from 14.4 million in the 2019 calendar year to 15.5 million in 2023 (MMPC, 0.32%; 95% confidence interval [CI], 0.12–0.52%); estimated emissions increased from 217 510 t to 246 934 t CO_2_e (MMPC, 0.46%; 95% CI, 0.22–0.71%). The increased dispensing of inhalers was primarily caused by the increased dispensing of high emissions inhalers, from 8.2 million to 9.2 million (MMPC, 0.43%; 95% CI, 0.22–0.65%); the dispensing of low emissions inhaler increased from 6.2 million to 6.3 million (MMPC, 0.15%; 95% CI, 0.01–0.29%). In 2019, 56.9% of dispensed PBS‐subsidised inhalers were high emission inhalers, and 59.5% in 2023; in each year, they accounted for about 98% of total estimated PBS‐subsidised inhaler‐related emissions (Box 1; Box 2). The sensitivity analysis yielded similar results (Supporting Information, table 2).

Box 1 **Pharmaceutical Benefits Scheme‐subsidised dispensing of respiratory inhalers and estimated associated carbon emissions, Australia, 2019–2023, by year**

**Table:** 

	Low emissions inhalers*			High emissions inhalers^†^			All inhalers		
Year	Inhalers dispensed	Inhalers/1000 population^‡^	CO_2_e (t)	Inhalers dispensed	Inhalers/1000 population^‡^	CO_2_e (t)	Inhalers dispensed	Inhalers/1000 population^‡^	CO_2_e (t)
2019	6 217 094 (43.1%)	213	4681 (2.2%)	8 222 259 (56.9%)	301	212 830 (97.8%)	14 439 353	514	217 510
2020	6 642 484 (41.8%)	222	5090 (2.1%)	9 242 574 (58.2%)	331	242 270 (97.9%)	15 885 058	553	247 360
2021	6 325 195 (42.4%)	207	4854 (2.1%)	8 582 617 (57.6%)	303	225 880 (97.9%)	14 907 812	510	230 734
2022	6 670 531 (40.8%)	215	5266 (2.0%)	9 676 555 (59.2%)	338	255 227 (98.0%)	16 347 086	553	260 493
2023^§^	6 290 153 (40.5%)	198	4881 (2.0%)	9 241 921 (59.5%)	312	242 053 (98.0%)	15 532 073	509	246 934
MMPC, 2019–2023 (95% CI)^¶^	0.15% (0.01 to 0.29%)	–0.02% (–0.17 to 0.13%)	0.21% (0.05 to 0.36%)	0.43% (0.22 to 0.65%)	0.27%. (0.05 to 0.49%)	0.46% (0.24 to 0.67%)	0.32% (0.12 to 0.52%)	0.17% (–0.05 to 0.38%)	0.46% (0.22 to 0.71%)

CI = confidence interval; CO_2_e = carbon dioxide equivalent; MMPC = mean monthly percentage change.

* Dry powder inhalers and soft mist inhalers.

† Pressurised metered dose inhalers and breath‐actuated inhalers.

‡ Age‐standardised to the Australian standard population (2010).^3^

§ Data available for January to November; totals and rates were multiplied by 1.099 to provide estimate for year.

¶ Data for March 2020 were replaced by mean data for March 2019 and March 2021 to reduce the influence of coronavirus disease 2019 (COVID‐19) pandemic on the estimates.

Box 2 Pharmaceutical Benefits Scheme (PBS)‐subsidised dispensing of respiratory inhalers and estimated carbon emissions, Australia, 2019–2023, by inhaler type and month*

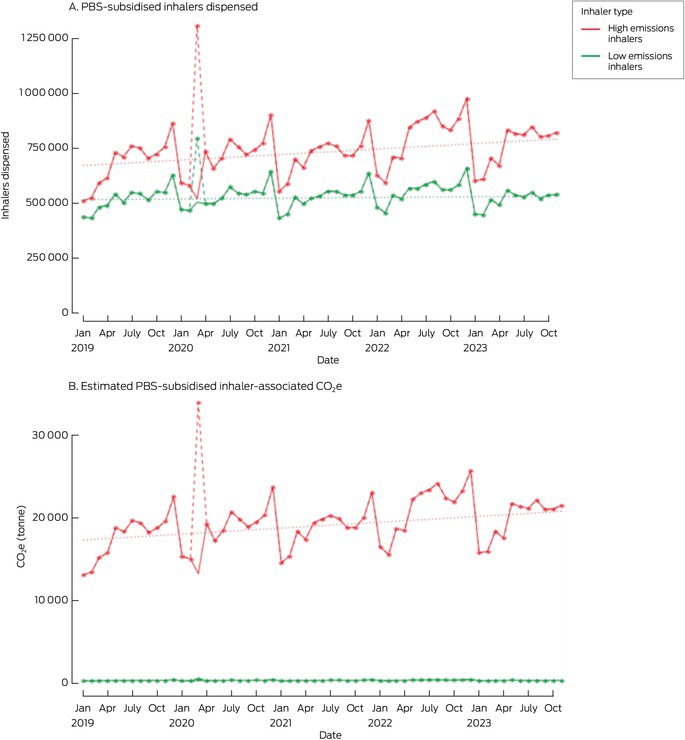

CO_2_e = carbon dioxide equivalent.

* Solid line: raw data; dotted line: linear trend, 2019–2023; dashed line (March 2020: coronavirus disease 2019‐related divergence from normal dispensing): unadjusted raw data that were replaced by mean of values for March 2019 and March 2021.

Estimated emissions were greatest for short‐acting β_2_ agonist inhalers (4.8–5.5 million dispensed per year; 2023: 98% in high emissions inhalers; 140 558 t CO_2_e, 57% of all inhaler‐related emissions) and combined inhaled corticosteroid/long‐acting β_2_ agonist inhalers (5.6–6.4 million dispensed per year; 2023: 49% in high emissions inhalers; 83 491 t CO_2_e, 34% of all inhaler‐related emissions) (Supporting Information, table 3 and figure 1).

PBS‐subsidised inhaler dispensing rates were higher in non‐metropolitan (2023: 704 per 1000 population) than in metropolitan areas (2023: 450 per 1000 population), but estimated total emissions were higher in metropolitan areas because of the larger numbers of inhalers dispensed (2023: metropolitan, 155 878 t CO_2_e; non‐metropolitan: 91 010 t CO_2_e). Estimated emissions increased in non‐metropolitan areas during 2019–2023 for both low emissions inhalers (MMPC, 0.29%; 95% CI, 0.17–0.41%) and high emissions inhalers (MMPC, 0.53%; 95% CI, 0.34–0.73%); in metropolitan areas, they increased for high emissions inhalers only (MMPC, 0.43%; 95% CI, 0.16–0.72%) (Supporting Information, table 3).

PBS‐subsidised inhaler dispensing rates were highest in Tasmania (2023: 693 per 1000 population) and South Australia (2023: 632 per 1000 population) and lowest in the Northern Territory (355 per 1000 population) and Western Australia (383 per 1000 population). The proportions of high emissions inhalers dispensed were largest in South Australia (63–65%) and the Northern Territory (59–64%) and smallest in the Australian Capital Territory (53–57%) and Western Australia (52–56%). Total estimated emissions were highest in New South Wales (2023: 78 500 t CO_2_e) and Victoria (2023: 62 622 t CO_2_e). Emissions increased in all states and territories between 2019 and 2023, primarily because of the increased dispensing of high emissions inhalers; MMPCs ranged from 0.31 (95% CI, 0.04–0.58%) in New South Wales to 0.64 (95% CI, 0.35–0.93%) in Victoria (Supporting Information, table 3).

In 2023, the PBS‐subsidised inhaler dispensing rate was 195 per 1000 people aged 0–19 years (high emissions inhalers, 92%) and 2054 per 1000 people aged 80 years or older (high emissions inhalers, 50%) (Box 3). The age group dispensed the largest proportion of PBS‐subsidised inhalers were people aged 60–79 years (6.4–7.1 million/year; 43–45% of all PBS‐subsidised inhalers dispensed; high emissions inhalers, 50–54%), contributing 100 688t CO_2_e in 2023 (about 41% of all emissions). Increases in PBS‐subsidised high emissions inhaler dispensing rates (MMPC, 0.34%; 95% CI, 0.03–0.66%) and emissions (MMPC, 0.61%; 95% CI, 0.30–0.91%) were greatest for people aged 80 years or older. PBS‐subsidised dispensing numbers and rates were higher for female than male users (2023: 549 *v* 462 per 1000 population); the proportions of high emissions inhalers dispensed were larger for female than male users (58–60% *v* 55–58%), and the increase in high emissions inhaler‐related emissions was greater (MMPC, 0.50%; 95% CI, 0.23–0.78% *v* 0.44%; 95% CI, 0.19–0.69%) (Supporting Information, table 3).

Box 3 Pharmaceutical Benefits Scheme‐subsidised dispensing of respiratory inhalers, Australia, 2019, 2021, and 2023: dispensing rates (per 1000 population) by demographic characteristics*

**Table:** 

	2019		2021		2023^†^	
Characteristic	Low emissions inhalers^‡^	High emissions inhalers^§^	Low emissions inhalers^‡^	High emissions inhalers^§^	Low emissions inhalers^‡^	High emissions inhalers^§^
All people	213	301	207	303	198	312
Area						
Metropolitan	176	259	172	260	171	279
Non‐metropolitan	259	361	280	401	282	422
State/territory						
Australian Capital Territory	199	245	206	254	211	245
New South Wales	227	322	212	311	200	313
Northern Territory	149	198	148	218	133	222
Queensland	222	303	220	307	207	312
South Australia	209	416	208	406	199	432
Tasmania	264	399	260	427	249	445
Victoria	203	281	200	292	195	313
Western Australia	180	202	177	221	167	216
Age group (years)						
0–19	15	198	13	180	16	179
20–39	65	132	67	143	65	136
40–59	201	294	202	319	197	323
60–79	747	735	703	717	658	774
80 or older	1133	954	1098	945	1036	1017
Gender						
Female	221	329	215	331	208	341
Male	205	268	197	272	185	277
Inhaler class						
Inhaled corticosteroid	7	32	6	30	9	26
Inhaled corticosteroid/long‐acting β_2_ agonist	113	88	109	94	102	99
Inhaled corticosteroid/long‐acting muscarinic antagonist/long‐acting β_2_ agonist	10	—	17	1	25	9
Long‐acting β_2_ agonist	63	—	52	—	41	—
Long‐acting muscarinic antagonist/long‐acting β_2_ agonist	21	—	21	—	18	—
Short‐acting β_2_ agonist	—	176	1	172	3	170
Short‐acting muscarinic antagonist	—	6	—	7	—	8

* Rates are age‐standardised to the Australian standard population (2010).^3^ For space reasons, data for three years are included here; the dispensing rates for all five years are included in the Supporting Information, table 3.

† Data available for January to November; totals and rates were multiplied by 1.099 to provide estimate for year.

‡ Dry powder inhalers and soft mist inhalers.

§ Pressurised metered dose inhalers and breath‐actuated inhalers.

Limitations to our study include the omission of over‐the‐counter sales from our detailed analysis, some missing data, and the use of United Kingdom data^2^ for emission estimates. Based on the findings of another study,^4^ we estimate that total emissions from both over‐the‐counter short‐acting β_2_ agonist inhalers and all dispensed PBS‐subsidised prescription inhalers in Australia in 2019 were 437 867 t CO_2_e, or 17.3 kg CO_2_e per person (details: Supporting Information, part 1). In Great Britain, 61.1 million inhalers contributed 20.4 kg CO_2_e per person in 2021,^5^ in Sweden 4.8 million inhalers (in 2017)^6^ contributed 2.5 kg CO_2_e emissions per person per year (in 2018–19).^7^

In the first comprehensive estimate of carbon emissions for PBS‐subsidised inhalers in Australia, we found differences in dispensing according to the demographic characteristics of inhaler users. To better support efforts to reduce respiratory inhaler emissions as part of Australian health care decarbonisation plans,^1^ we recommend routine collecting and reporting of over‐the‐counter inhaler sales data, and that these data are made freely available to complement PBS‐subsidised dispensing data. Our findings could inform targeted interventions for reducing the use of high emission inhalers in Australia and updates to clinical practice guidelines.

## Open access

Open access publishing facilitated by University of Canberra, as part of the Wiley – University of Canberra agreement via the Council of Australian University Librarians.

## Competing interests

Luise Kazda was reimbursed by Asthma Australia for travel expenses to attend a round table meeting on sustainable asthma care.

## Data sharing

The de‐identified data we analysed are not publicly available and will not be shared, as we do not have permission from the data custodian or ethics approval to do so.

## Supporting information


Supplementary methods and results
