# Supplementary material for: Estimated carbon emissions for PBS‐subsidised prescription respiratory inhalers, Australia, 2019–2023: a descriptive analysis
Source: Med J Aust. 2025 Jun 26;223(4):214–7. doi: 10.5694/mja2.52715 (PMC12358100; doi:10.5694/mja2.52715)
Supplement: Supplementary file 1 — Supplementary methods and results [file MJA2-223-214-s001.pdf]

MJA
The Medical Journal of Australia

Supporting Information

Supplementary methods and results

This appendix was part of the submitted manuscript and has been peer reviewed.
It is posted as supplied by the authors.

Appendix to: Kazda L, Barratt AL, Docking S, et al. Estimated carbon emissions for PBS-subsidised prescription respiratory inhalers, Australia, 2019–2023: a descriptive analysis. *Med J Aust* 2025; doi: 10.5694/mja2.52715.

1. Supplementary methods

Carbon footprint

As previously done by others[1], we based our emissions estimates for each inhaler on established carbon footprint estimates compiled for the United Kingdom National Health Service[2]. Australian inhalers listed on the Pharmaceutical Benefit Scheme (PBS) were matched to their United Kingdom equivalent and an emissions estimate (in grams of carbon dioxide equivalent (CO_{2e}) emissions) tabulated for each inhaler (table 1).

Population estimates

For age-standardised rates we used data from the Australian Bureau of Statistics to determine annual total population numbers for Australia[3] and the Australian Standard Population from 2010 as the reference population[4].

Missing data

To ensure de-identification was maintained, cell suppression was applied by Services Australia to the dataset when five or fewer (but at least one) scripts were dispensed for an item code for a month, patient gender, age group, and location. Overall, 181,683 of 2,049,974 rows (8.9%) were suppressed and numbers of scripts missing. We assumed that the number of missing scripts dispensed was 3 for each suppressed cell (median of the possible number of scripts for a suppressed cell). This yielded a total number of scripts dispensed of 61,759,923. For comparison, replacing all missing script values with 1 (the lowest possible value) would yield 61,396,557 scripts (0.6% fewer), replacing them with 5 (the highest possible value) would yield 62,123,289 scripts (0.6% more).

Statistical analysis

We undertook descriptive statistical analysis to determine the total number of inhalers dispensed (by type: high emissions or low emissions inhalers) for each month and by year. We aggregated emissions for all inhalers and summarised the total estimated carbon emissions for each month and year by inhaler type. We also summarised age-standardised rates of inhaler dispensing per 1,000 population by type for each calendar year and by patient location (area [metropolitan, non-metropolitan] and jurisdiction), gender, and age, as well as by inhaler class. To enable annual comparisons despite missing data for December 2023 (the dataset was provided in December 2023), we multiplied aggregated data from January to November 2023 by 1.099 to provide a twelve month estimate for these analyses. This multiplication factor was derived from the mean December weight for inhalers dispensed in the previous four years.

We then conducted time series analysis using the monthly values and adjusting for autocorrelation to plot a regression line showing an overall trend (without seasonality) for each stratified rate and total numbers of inhalers and estimated carbon emissions. We modelled mean monthly percentage changes (MMPC) using joinpoint regression analysis. Joinpoint Regression Program 5.0.2 was used to model mean monthly percentage changes; all other analyses and visualisation were performed using SAS/STAT 15.1 (SAS 9.4M6).

Coronavirus disease 2019 (COVID-19) pandemic outliers

March 2020 inhaler dispensing data presents an extreme outlier in the dataset due to the Covid-19 pandemic and associated consumer stockpiling of inhalers[5,6]. In graphic visualisation we present this datapoint as dashed together with a solid line alternative through a datapoint for March 2020 based on the mean dispensing from March 2019 and March 2021. For trend analyses we used this mean data to replace actual March 2020 dispensing to minimise impact of this extreme outlier on trends.

Pharmaceutical Benefit Scheme (PBS) item codes

In Australia, eligible pharmaceuticals are subsidised and dispensed to all citizens, permanent residents and visitors with reciprocal health care agreements under the PBS. War veterans (about 2.8% of people aged 15 years or older) are eligible for the Repatriation Pharmaceutical Benefits Scheme (RPBS); data from this scheme are not considered in our analyses[7]. Respiratory inhalers available on prescription in Australia are listed under the PBS and their dispensing is recorded by Services Australia. We manually searched all item codes currently listed under "R03 – Drugs for Obstructive Airway Diseases" and the "PBS Archives Summary of Changes" from January 2019 until December 2023 for any added or deleted item codes relating to respiratory inhalers[8]. For each PBS item code, we recorded the brand names, dose, actuations (number of puffs), maximum quantity, and active ingredients. The final list of PBS item codes submitted to Services Australia for data release and for inclusion in the analysis can be found in table 1. This list includes all items listed on the PBS, even those where the co-payment threshold is not reached, i.e. the patient pays the full amount.

Number of scripts and number of inhalers

The number of scripts dispensed for each item code per month (based on date of supply) was provided by Service Australia. Whilst most scripts for inhalers stipulate a maximum quantity of one inhaler to be dispensed at the same time, there are some item codes for which two inhalers can be dispensed simultaneously. These were identified (table 1) and, for most of them, we assumed that for these codes two inhalers (i.e. the maximum) were dispensed for each script. For short-acting beta$_2$ agonist (SABA) inhaler scripts, our main analysis is based on the assumption that a mean of 1.5 inhalers were dispensed per script because it is prescribed as reliever medication (requirement can vary substantially depending on user) and because of shortages during the Covid-19 pandemic that may have led to some pharmacists only dispensing one SABA inhaler at a time. A conservative assumption of one SABA inhaler dispensed per prescription is explored in a sensitivity analysis. To derive the number of inhalers dispensed per month for each item code, the number of scripts was multiplied by the maximum number of items per script (capped at 1.5 for SABA inhalers). Over the five-year time period, an overall 22.3% higher number of assumed inhalers were dispensed than number of scripts.

User demographic characteristics

User demographic characteristics supplied by Services Australia were used to stratify scripts dispensed by location of residence (address at the end of each year) into jurisdiction (state/territory) and area (metropolitan/non-metropolitan) (based on the Australia Bureau of Statistics (ABS) SA4 classification[9]), by age group (20-year groups) and by gender, reported by Services Australia in binary categories (female/male) and referring to "the way a person feels, presents and is recognised in the community".[10]

Inhaler type

We categorised inhalers into four types based on PBS item descriptor: pressurised metered dose inhalers, breath-actuated inhalers, dry powder inhalers, and soft mist inhalers. We circulated this list to a pharmacist and two clinicians for verification. As very few breath-actuated and soft mist inhalers were dispensed, we defined a binary variable combining breath-actuated inhalers with pressurised metered dose inhalers as high emissions inhalers, and soft mist inhalers with dry powder inhalers as low emissions inhalers.

Inhaler class

We used the National Asthma Council's "Asthma & COPD Medications" chart[11] to link the active ingredients for each PBS item number to the class of drug contained in the inhaler. Inhalers were grouped into seven classes: inhaled corticosteroid (ICS), combined inhaled corticosteroid/ long-acting beta$_2$ agonist (ICS/LABA), combined inhaled corticosteroid/ long-acting muscarinic antagonist/ long-acting beta$_2$ agonist (ICS/LAMA/LABA), long-acting beta$_2$ agonist (LABA), combined long-acting muscarinic antagonist/long-acting beta$_2$ agonist

(LAMA/LABA), short-acting beta$_2$ agonist (SABA) and short-acting muscarinic antagonist (SAMA) inhalers.[12] We circulated this list to a pharmacist and two clinicians for verification (table 1).

Methods for carbon dioxide equivalent emissions estimate calculation including over-the-counter sales

Removing the estimated carbon emissions of PBS-subsidised SABA inhalers (134,763t CO_2e) from our estimate of total PBS CO_2e emission for 2019 leaves 82,747t CO2e; adding Alzaabi's estimate for all (PBS and over-the-counter (OTC) SABA inhalers for 2019 (355,114 tonnes) gives 437,861t CO_2e emissions.[13]

References

1. Wurzel DF, Montgomery BD, Anderson N, et al. Environmental impact of inhaled medicines: A Thoracic Society of Australia and New Zealand position statement. *Respirology* 2025;30:101-12.
2. PrescQIPP Community Interest Company. Inhaler Carbon Footprint, attachment 1 - Inhaler carbon footprint data London: PrescQIPP Consumer Interest Company; 2021 [updated October 2021. Available from: https://www.prescqipp.info/media/5tsdrgha/attachment-1-inhaler-carbon-footprint-data-235.xlsx accessed 27/02/2025.
3. Australian Bureau of Statistics. National, state and territory population Canberra: Australian Bureau of Statistics; updated December 2024. Available from: https://www.abs.gov.au/statistics/people/population/national-state-and-territory-population/latest-release accessed 27/02/2025.
4. Australian Bureau of Statistics. Feature Article: Which population to use for age standardisation? Canberra: Australian Bureau of Statistics; 2013 [updated December 2013. Available from: https://www.abs.gov.au/ausstats/abs@.nsf/Lookup/3101.0Feature+Article1Mar%202013 accessed 01/08/2024 2024.
5. Bowden T. Coronavirus sees pharmacies run out of critical medication, endangering the lives of people with chronic illnesses 2020 updated 24/03/2020. Available from: https://www.abc.net.au/news/2020-03-24/coronavirus-panic-buying-sees-shortage-of-vital-medicine/12081436 accessed 27/02/2025.
6. Mahase E. Covid-19: Increased demand for steroid inhalers causes “distressing” shortages. *BMJ* 2020;369:m1393.
7. Mellish L, Karanges EA, Litchfield MJ, et al. The Australian Pharmaceutical Benefits Scheme data collection: a practical guide for researchers. *BMC Res Notes* 2015;8:634.
8. Department of Health and Aged Care. The Pharmaceutical Benefits Scheme (PBS) Canberra: Australian Government, Department of Health and Aged Care; [Available from: https://www.pbs.gov.au/pbs/home accessed 01/08/2024.
9. Australian Bureau of Statistics. Statistical Area Level 4 updated July 2021. Available from: https://www.abs.gov.au/statistics/standards/australian-statistical-geography-standard-asgs-edition-3/jul2021-jun2026/main-structure-and-greater-capital-city-statistical-areas/statistical-area-level-4 accessed 27/002/2025.
10. Services Australia. Gender definitions 2023 updated December 2023. Available from: https://www.servicesaustralia.gov.au/updating-your-gender-details-with-us accessed 26/02/2025.
11. National Asthma Council. Asthma and COPD Medications Chart Melbourne: National Asthma Council Australia; 2023 updated 2024. Available from: https://files.nationalasthma.org.au/resources/NAC038-Asthma-COPD-Medications-Chart-2024_A4_WEB.pdf accessed 27/02/2025.
12. Mosnaim G. Asthma in Adults. *N Engl J Med* 2023;389:1023-31.
13. Alzaabi A, Bell JP, Montero-Arias F, et al. Greenhouse Gas Emissions from Respiratory Treatments: Results from the SABA CARBON International Study. *Adv Ther* 2023;40:4836-56.

Table 1. Pharmaceutical Benefits Scheme-subsidised inhalers and their estimated emissions per inhaler

Item Code	Brand Names	Dose (micrograms)	Actuations	Maximum quantity/ script	Active ingredient 1	Active ingredient 2	Active ingredient 3	Type	Class	Indicative emissions (g CO_2e)[1]
08408M	Qvar 50 Autohaler	50	200	1	Beclometasone			BAI	ICS	20,350
08409N	Qvar 100 Autohaler	100	200	1	Beclometasone			BAI	ICS	20,350
08406K	Qvar 50	50	200	1	Beclometasone			MDI	ICS	20,350
08407L	Qvar 100	100	200	1	Beclometasone			MDI	ICS	20,350
02070Y	Pulmicort Turbuhaler	100	200	1	Budesonide			DPI	ICS	1,400
02071B	Pulmicort Turbuhaler	200	200	1	Budesonide			DPI	ICS	1,400
02072C	Pulmicort Turbuhaler	400	200	1	Budesonide			DPI	ICS	1,700
08853Y	Alvesco Inhaler	80	120	1	Ciclesonide			MDI	ICS	12,210
08854B	Alvesco Inhaler	160	120	1	Ciclesonide			MDI	ICS	12,210
11719T	Arnuity Ellipta	100	30	1	Fluticasone Furoate			DPI	ICS	765
11729H	Arnuity Ellipta	200	30	1	Fluticasone Furoate			DPI	ICS	765
08147T	Axotide Junior Accuhaler<br>Flixotide Junior Accuhaler	100	60	1	Fluticasone Propionate			DPI	ICS	833
08148W	Axotide Accuhaler<br>Flixotide Accuhaler	250	60	1	Fluticasone Propionate			DPI	ICS	833
08149X	Flixotide Accuhaler	500	60	1	Fluticasone Propionate			DPI	ICS	833
08345F	Axotide<br>Fluticasone Cipla Inhaler<br>Flixotide	125	120	1	Fluticasone Propionate			MDI	ICS	19,277
08346G	Axotide<br>Fluticasone Cipla Inhaler<br>Flixotide	250	120	1	Fluticasone Propionate			MDI	ICS	19,277
08516F	Axotide Junior<br>Flixotide Junior	50	120	1	Fluticasone Propionate			MDI	ICS	19,277
12183F	Fostair Inhaler	100/6	120	1	Beclometasone	Formoterol		MDI	ICS/LABA	11,249
13205B	Fostair	200/6	120	1	Beclometasone	Formoterol		MDI	ICS/LABA	14,153
08625Y	Symbicort Turbuhaler 200/6<br>BiResp Spiromax<br>DuoResp Spiromax<br>Rilast Turbuhaler 200/6	200/6	120	1	Budesonide	Formoterol		DPI	ICS/LABA	800
08750M	Symbicort Turbuhaler 400/12<br>BiResp Spiromax<br>DuoResp Spiromax	400/12	60	2	Budesonide	Formoterol		DPI	ICS/LABA	1,050
08796Y	Symbicort Turbuhaler 100/6	100/6	120	1	Budesonide	Formoterol		DPI	ICS/LABA	580

Item Code	Brand Names	Dose (micrograms)	Actuations	Maximum quantity/ script	Active ingredient 1	Active ingredient 2	Active ingredient 3	Type	Class	Indicative emissions (g CO_2e)[1]
11273H	Symbicort Turbuhaler 200/6<br>BiResp Spiromax<br>DuoResp Spiromax<br>Rilast Turbuhaler 200/6	200/6	120	1	Budesonide	Formoterol		DPI	ICS/LABA	800
11301T	Symbicort Turbuhaler 400/12<br>BiResp Spiromax<br>DuoResp Spiromax	400/12	60	2	Budesonide	Formoterol		DPI	ICS/LABA	1,050
12029D	BiResp Spiromax<br>DuoResp Spiromax	200/6	120	2	Budesonide	Formoterol		DPI	ICS/LABA	630
12041R	Symbicort Turbuhaler 200/6<br>BiResp Spiromax<br>DuoResp Spiromax<br>Rilast Turbuhaler 200/6	200/6	120	1	Budesonide	Formoterol		DPI	ICS/LABA	800
12093L	Symbicort Turbuhaler 200/6<br>BiResp Spiromax<br>DuoResp Spiromax<br>Rilast Turbuhaler 200/6	200/6	120	1	Budesonide	Formoterol		DPI	ICS/LABA	800
12101X	Symbicort Turbuhaler 100/6	100/6	120	1	Budesonide	Formoterol		DPI	ICS/LABA	580
13258T	DuoResp Spiromax<br>Rilast Turbohaler 400/12<br>Symbicort Turbohaler 400/12	400/12	60	2	Budesonide	Formoterol		DPI	ICS/LABA	630
10015D	Symbicort Rapihaler 100/3<br>Rilast Rapihaler 100/3	100/3	120	2	Budesonide	Formoterol		MDI	ICS/LABA	34,400
10018G	Symbicort Rapihaler 200/6<br>Rilast Rapihaler 200/6	200/6	120	2	Budesonide	Formoterol		MDI	ICS/LABA	34,400
10024N	Symbicort Rapihaler 50/3	50/3	120	2	Budesonide	Formoterol		MDI	ICS/LABA	34,400
12042T	Symbicort Rapihaler 100/3<br>Rilast Rapihaler 100/3	100/3	120	2	Budesonide	Formoterol		MDI	ICS/LABA	34,400
12082X	Symbicort Rapihaler 200/6<br>Rilast Rapihaler 200/6	200/6	120	2	Budesonide	Formoterol		MDI	ICS/LABA	34,400
12089G	Symbicort Rapihaler 100/3<br>Rilast Rapihaler 100/3	100/3	120	2	Budesonide	Formoterol		MDI	ICS/LABA	34,400
12100W	Symbicort Rapihaler 50/3	50/3	120	2	Budesonide	Formoterol		MDI	ICS/LABA	34,400
11124L	Breo Ellipta 100/25	100/25	30	1	Fluticasone Furoate	Vilanterol		DPI	ICS/LABA	754
11129R	Breo Ellipta 200/25	200/25	30	1	Fluticasone Furoate	Vilanterol		DPI	ICS/LABA	754
02827T	Flutiform 50/5	5/50	120	1	Fluticasone Propionate	Formoterol		MDI	ICS/LABA	36,500
10007Q	Flutiform 125/5	5/125	120	1	Fluticasone Propionate	Formoterol		MDI	ICS/LABA	36,500

Item Code	Brand Names	Dose (micrograms)	Actuations	Maximum quantity/ script	Active ingredient 1	Active ingredient 2	Active ingredient 3	Type	Class	Indicative emissions (g CO_2e)[1]
10008R	Flutiform 250/10	10/250	120	1	Fluticasone Propionate	Formoterol		MDI	ICS/LABA	36,500
08430Q	Pavtide Accuhaler 100/50<br>Seretide Accuhaler 100/50	50/100	60	1	Fluticasone Propionate	Salmeterol		DPI	ICS/LABA	898
08431R	Fluticasone + Salmeterol Ciplahaler 250/50<br>Pavtide Accuhaler 250/50<br>Seretide Accuhaler 250/50	50/250	60	1	Fluticasone Propionate	Salmeterol		DPI	ICS/LABA	898
08432T	Pavtide Accuhaler 500/50<br>Seretide Accuhaler 500/50<br>Fluticasone Salmeterol Ciphaler 500/50	50/500	60	1	Fluticasone Propionate	Salmeterol		DPI	ICS/LABA	898
08517G	Pavtide MDI 50/25<br>Seretide MDI 50/25	25/50	120	1	Fluticasone Propionate	Salmeterol		MDI	ICS/LABA	19,485
08518H	Evocair MDI[3]<br>Fluticasone + Salmeterol Cipla 125/25<br>Pavtide<br>SalplusF Inhaler 125/25<br>Seroflo 125/25<br>Seretide MDI 125/25	25/125	120	1	Fluticasone Propionate	Salmeterol		MDI	ICS/LABA	17,878
08519J	Evocair MDI[3]<br>Fluticasone + Salmeterol Cipla 250/25<br>Pavtide<br>SalplusF Inhaler 250/25<br>Seroflo 250/25<br>Seretide MDI 250/25	25/250	120	1	Fluticasone Propionate	Salmeterol		MDI	ICS/LABA	17,878
12269R	Atectura Breezhaler	125/62.5	30	1	Indacaterol	Mometasone		DPI	ICS/LABA	401
12279G	Atectura Breezhaler	125/260	30	1	Indacaterol	Mometasone		DPI	ICS/LABA	401
12289T	Atectura Breezhaler	125/127.5	30	1	Indacaterol	Mometasone		DPI	ICS/LABA	401
12468F	Trimbow	100/6/10	120	1	Beclometasone	Formoterol	Glycopyrronium	MDI	ICS/LAMA/LABA	14,203
13200R	Trimbow	200/6/10	120	1	Beclometasone	Formoterol	Glycopyrronium	MDI	ICS/LAMA/LABA	14,203
12672Y	Breztri Aerosphere	160/7.2/5	120	1	Budesonide	Formoterol	Glycopyrronium	MDI	ICS/LAMA/LABA	13,500

Item Code	Brand Names	Dose (micrograms)	Actuations	Maximum quantity/ script	Active ingredient 1	Active ingredient 2	Active ingredient 3	Type	Class	Indicative emissions (g CO_2e)[1]
11379X	Trelegy Ellipta	100/62.5/25	30	1	Fluticasone Furoate	Umeclidinium	Vilanterol	DPI	ICS/LAMA/LABA	765
12917W	Trelegy Ellipta	200/62.5/25	30	1	Fluticasone Furoate	Umeclidinium	Vilanterol	DPI	ICS/LAMA/LABA	765
12295D	Enerzair Breezhaler	114/46/136	30	1	Indacaterol	Glycopyrronium	Mometasone	DPI	ICS/LAMA/LABA	382
12298G	Enerzair Breezhaler	114/46/68	30	1	Indacaterol	Glycopyrronium	Mometasone	DPI	ICS/LAMA/LABA	382
10124W	Bretaris Genuair	322	60	1	Aclidinium			DPI	LABA	520
08136F	Foradile	12	60	1	Formoterol			DPI	LABA	1,125
08239P	Oxis Turbuhaler	6	60	1	Formoterol			DPI	LABA	360
08240Q	Oxis Turbuhaler	12	60	1	Formoterol			DPI	LABA	370
10059K	Seebri Breezhaler	50	30	1	Glycopyrronium			DPI	LABA	563
05134F	Onbrez Breezhaler	150	30	1	Indacaterol			DPI	LABA	563
05137J	Onbrez Breezhaler	300	30	1	Indacaterol			DPI	LABA	563
08141L	Serevent Accuhaler	50	60	1	Salmeterol			DPI	LABA	732
08626B	Spiriva Handihaler	18	30	1	Tiotropium			DPI	LABA	282
11892X	Braltus Zonda	13	30	1	Tiotropium			DPI	LABA	563
10509D	Spiriva Respimat	2.5	60	1	Tiotropium			SMI	LABA	775
11043F	Spiriva Respimat	2.5	60	1	Tiotropium			SMI	LABA	775
11629C	Spiriva Respimat	2.5	60	1	Tiotropium			SMI	LABA	775
10187E	Incruse Ellipta	62.5	30	1	Umeclidinium			DPI	LABA	731
10565C	Brimica Genuair	340/12	60	1	Aclidinium	Formoterol		DPI	LAMA/LABA	550
10156M	Ultibro Breezhaler	110/50	30	1	Indacaterol	Glycopyrronium		DPI	LAMA/LABA	563
10557P	Spiolto Respimat	2.5/2.5	60	1	Tiotropium	Olodaterol		SMI	LAMA/LABA	775
10188F	Anoro Ellipta	62.5/25	30	1	Umeclidinium	Vilanterol		DPI	LAMA/LABA	747
08354Q	Airomir Autohaler	100	200	2	Salbutamol			BAI	SABA	9,720
08288F	Ventolin CFC-Free[2]<br>Asmol CFC-Free<br>Zempreon CFC-Free	100	200	2	Salbutamol			MDI	SABA	28,262
12108G	Ventolin CFC-Free[2]<br>Asmol CFC-Free<br>Zempreon CFC-Free	100	200	2	Salbutamol			MDI	SABA	28,262
12109H	Ventolin CFC-Free[2]<br>Asmol CFC-Free<br>Zempreon CFC-Free	100	200	2	Salbutamol			MDI	SABA	28,262
02817G	Bricanyl Turbuhaler	500	120	2	Terbutaline			DPI	SABA	492
12267P	Bricanyl Turbuhaler	500	120	2	Terbutaline			DPI	SABA	492

Item Code	Brand Names	Dose (micrograms)	Actuations	Maximum quantity/ script	Active ingredient 1	Active ingredient 2	Active ingredient 3	Type	Class	Indicative emissions (g CO_2e)[1]
08671J	Atrovent Metered Aerosol	21	200	2	Ipratropium			MDI	SAMA	14,590

[1]All emissions based on PrescQIPP Community Interest Company data.[2]

[2]Emissions available only for Ventolin, it is assumed that Asmol and Zempreon have a similar value.

[3]Mean for Seretide (19,485g CO2e) and Seroflo (16,270g CO2e) used in the analysis.

BAI= breath actuated inhaler; CO_2e= carbon dioxide equivalent; DPI= dry powder inhaler; g=gram, ICS= inhaled corticosteroid; ICS/LABA= combined inhaled corticosteroid/ long-acting $beta_2$ agonist; ICS/LAMA/LABA= combined inhaled corticosteroid/ long-acting muscarinic antagonist/ long-acting $beta_2$ agonist; LABA= long-acting $beta_2$ agonist; LAMA/LABA= combined long-acting muscarinic antagonist / long-acting $beta_2$ agonist; MDI=metered dose inhaler; mgr=microgram; PBS= Pharmaceutical Benefits Scheme; SABA= short-acting $beta_2$ agonist; SAMA= short-acting muscarinic antagonist; SMI= soft mist inhaler

Supplementary results

Table 2. Sensitivity analysis: Pharmaceutical Benefits Scheme-subsidised respiratory inhalers dispensed per year and their carbon emissions (one SABA inhaler dispensed per script)

Year	Low emissions inhalers[1]			High emissions inhalers[2]			All inhalers		
	Inhalers dispensed	Inhalers/1000 population[3]	CO_2e (t)	Inhalers dispensed	Inhalers/1000 population[3]	CO_2e (t)	Inhalers dispensed	Inhalers/1000 population[3]	CO_2e (t)
2019	6,217,094 (48.4%)	213	4,681 (2.7%)	6,619,188 (51.6%)	242	167,908 (97.3%)	12,836,282	455	172,589
2020	6,642,484 (41.8%)	222	5,090 (2.6%)	7,425,022 (52.8%)	266	191,265 (97.4%)	14,067,506	488	196,356
2021	6,310,562 (47.2%)	206	4,846 (2.6%)	6,951,817 (52.4%)	246	180,084 (97.4%)	13,262,379	452	184,931
2022	6,634,196 (47.6%)	214	5,248 (2.5%)	7,888,603 (54.3%)	276	205,023 (97.5%)	14,522,799	490	210,271
2023[4]	6,258,124 (45.3%)	196	4,797 (2.4%)	7,561,566 (54.7%)	255	195,241 (97.6%)	13,819,690	451	200,038
MMPC[5] (95% CI)	0.14% (-0.01% to 0.29%)	-0.03% (-0.19% to 0.13%)	0.20%* (0.04% to 0.37%)	0.48%* (0.24% to 0.72%)	0.33%* (0.07% to 0.58%)	0.52%* (0.28% to 0.76%)	0.32%* (0.13% to 0.51%)	0.17% (-0.04% to 0.37%)	0.51%* (0.27% to 0.75%)

[1]includes dry powder inhalers and soft mist inhalers

[2]includes pressurised metred dose inhalers and breath actuated inhalers

[3]age-standardised to Australian Standard Population (2010)[4]

[4]data only available for January to November. Totals and rates were multiplied by 1.099 to provide an estimate for the whole year.

[5]MMPC= mean monthly percentage change (in %), a summary measure of the trend between January 2019 and December 2023, calculated as a mean of all monthly percentage changes over the entire time period.

* indicates that the MMPC is significantly different from zero. For these estimates data for March 2020 was replaced by the mean from March 2019 and March 2021 to minimise impact of Covid-19 pandemic

MMPC= mean monthly percentage change, CI=confidence interval; CO_2e= carbon dioxide equivalent; n=number; SABA= short-acting beta$_2$ agonist

Table 3a. Pharmaceutical Benefits Scheme-subsidised respiratory inhalers and estimated carbon emissions by demographic characteristics of users and inhaler class, 2019 and 2020

Year	2019						2020					
	Low emissions inhalers[1]			High emissions inhalers[2]			Low emissions inhalers[1]			High emissions inhalers[2]		
	dispensing rate	inhalers dispensed	CO_2e	dispensing rate	inhalers dispensed	CO_2e	dispensing rate	inhalers dispensed	CO_2e	dispensing rate	inhalers dispensed	CO_2e
Characteristic	(n/1,000)[3]	(n)	(t)	(n/1,000)[3]	(n)	(t)	(n/1,000)[3]	(n)	(t)	(n/1,000)[3]	(n)	(t)
**Area**												
Metropolitan	176	3,889,438	2,934	259	5,345,703	138,532	187	4,120,613	3,166	289	5,966,277	156,425
Non-metropolitan	259	2,327,656	1,747	361	2,876,556	74,298	282	2,521,871	1,924	413	3,276,298	85,845
**State/territory**												
Australian Capital Territory	199	86,229	66	245	104,805	2,613	214	96,063	74	283	124,960	3,173
New South Wales	227	2,130,318	1,611	322	2,823,311	73,088	230	2,216,277	1,704	345	3,080,699	80,761
Northern Territory	149	27,261	19	198	39,878	998	157	29,998	22	226	47,599	1,218
Queensland	222	1,283,740	962	303	1,654,210	43,247	233	1,387,829	1,058	327	1,828,954	48,315
South Australia	209	473,292	353	416	821,829	21,550	221	510,222	389	449	902,655	23,856
Tasmania	264	187,868	143	399	248,008	6,503	277	201,006	155	436	277,136	7,337
Victoria	203	1,502,388	1,128	281	1,961,606	50,639	215	1,625,971	1,244	316	2,252,591	58,958
Western Australia	180	525,985	399	202	568,550	14,189	190	575,112	444	252	727,942	18,650
**Age group**												
0-19 years	15	89,386	74	198	1,236,031	30,512	15	90,877	75	189	1,180,666	29,173
20-39 years	65	465,972	403	132	953,044	25,245	72	527,788	453	159	1,163,613	31,195
40-59 years	201	1,309,630	1,063	294	1,900,772	50,047	219	1,441,045	1,178	355	2,313,357	61,691
60-79 years	747	3,212,141	2,356	735	3,172,713	82,542	756	3,387,508	2,539	786	3,538,251	93,149
80 years and older	1,133	1,139,965	785	954	959,699	24,484	1,151	1,195,266	846	1,008	1,046,688	27,062
**Gender**												
Female	221	3,360,278	2,553	329	4,666,833	121,238	232	3,611,787	2,788	364	5,262,159	138,378
Male	205	2,856,816	2,128	268	3,555,426	91,591	211	3,030,697	2,302	294	3,980,415	103,892

Year	2019						2020					
	Low emissions inhalers[1]			High emissions inhalers[2]			Low emissions inhalers[1]			High emissions inhalers[2]		
	dispensing rate	inhalers dispensed	CO_2e	dispensing rate	inhalers dispensed	CO_2e	dispensing rate	inhalers dispensed	CO_2e	dispensing rate	inhalers dispensed	CO_2e
Characteristic	(n/1,000)[3]	(n)	(t)	(n/1,000)[3]	(n)	(t)	(n/1,000)[3]	(n)	(t)	(n/1,000)[3]	(n)	(t)
**Class**												
ICS	7	185,233	229	32	804,681	14,462	7	199,386	241	32	816,937	14,622
ICS/LABA	113	3,180,908	2,767	88	2,441,378	61,168	119	3,427,694	2,961	99	2,789,031	71,951
ICS/LAMA/LABA	10	303,557	232	-	-	-	15	463,849	355	-	-	-
LABA	63	1,914,325	1,016	-	-	-	59	1,868,990	1,056	-	-	-
LAMA/LABA	21	633,071	437	-	-	-	22	682,565	478	-	-	-
SABA	-	-	-	176	4,809,212	134,763	-	-	-	195	5,452,656	153,013
SAMA	-	-	-	6	166,988	2,436	-	-	-	6	183,950	2,684
**Total**	213	6,217,094	4,681	301	8,222,259	212,829	222	6,642,484	5,090	331	9,242,574	242,270

[1]includes dry powder inhalers and soft mist inhalers

[2]includes pressurised metred dose inhalers and breath actuated inhalers

[3]age-standardised to Australian Standard Population (2010)[4]

Abbreviations: MMPC= mean monthly percentage change, CI=confidence interval; CO_2e= carbon dioxide equivalent; ICS= inhaled corticosteroid; ICS/LABA= combined inhaled corticosteroid/ long-acting $beta_2$ agonist; ICS/LAMA/LABA= combined inhaled corticosteroid/ long-acting muscarinic antagonist/ long-acting $beta_2$ agonist; LABA= long-acting $beta_2$ agonist; LAMA/LABA= combined long-acting muscarinic antagonist / long-acting $beta_2$ agonist; n=number; SABA= short-acting $beta_2$ agonist; SAMA= short-acting muscarinic antagonist

Table 3b. Pharmaceutical Benefits Scheme-subsidised respiratory inhalers and estimated carbon emissions by demographic characteristics of users and inhaler class, 2021 and 2022

Year	2021						2022					
	Low emissions inhalers[1]			High emissions inhalers[2]			Low emissions inhalers[1]			High emissions inhalers[2]		
	dispensing rate	inhalers dispensed	CO_2e	dispensing rate	inhalers dispensed	CO_2e	dispensing rate	inhalers dispensed	CO_2e	dispensing rate	inhalers dispensed	CO_2e
Characteristic	(n/1,000)[3]	(n)	(t)	(n/1,000)[3]	(n)	(t)	(n/1,000)[3]	(n)	(t)	(n/1,000)[3]	(n)	(t)
**Area**												
Metropolitan	172	3,818,232	2,941	260	5,390,775	141,695	184	4,061,587	3,244	300	6,172,308	162,984
Non-metropolitan	280	2,506,963	1,913	401	3,191,842	84,185	293	2,608,945	2,022	440	3,504,248	92,243
**State/ Territory**												
Australian Capital Territory	206	94,787	73	254	114,378	2,909	227	107,097	88	272	124,697	3,203
New South Wales	212	2,076,011	1,598	311	2,813,423	73,953	218	2,159,522	1,704	345	3,133,317	82,414
Northern Territory	148	29,418	22	218	46,869	1,207	153	31,362	25	233	51,201	1,315
Queensland	220	1,349,927	1,030	307	1,750,147	46,459	225	1,420,041	1,103	334	1,950,419	51,928
South Australia	208	492,146	375	406	832,211	22,063	214	511,446	394	455	941,758	25,013
Tasmania	260	194,919	151	427	276,429	7,350	269	202,895	163	457	300,676	7,963
Victorie	200	1,535,120	1,177	292	2,093,287	55,136	214	1,657,608	1,329	342	2,463,694	65,198
Western Australia	177	552,849	428	221	655,818	16,801	181	580,519	460	234	710,730	18,191
**Age group**												
0-19 years	13	80,940	66	180	1,123,042	27,853	17	104,527	89	219	1,377,416	34,270
20-39 years	67	483,206	409	143	1,033,867	27,942	76	553,662	485	161	1,166,334	31,851
40-59 years	202	1,334,858	1,087	319	2,088,609	56,005	217	1,440,305	1,206	350	2,307,904	62,220
60-79 years	703	3,247,137	2,447	717	3,322,445	87,744	708	3,356,592	2,591	781	3,710,380	97,941
80 years and older	1,098	1,179,055	846	945	1,014,654	26,337	1,098	1,215,446	896	1,007	1,114,522	28,946
**Gender**												
Female	215	3,433,757	2,649	331	4,855,158	128,247	229	3,689,296	2,933	372	5,524,463	146,450
Male	197	2,891,438	2,205	272	3,727,459	97,633	200	2,981,236	2,333	299	4,152,093	108,777
**Class**												

Year	2021						2022					
	Low emissions inhalers[1]			High emissions inhalers[2]			Low emissions inhalers[1]			High emissions inhalers[2]		
	dispensing rate	inhalers dispensed	CO_2e	dispensing rate	inhalers dispensed	CO_2e	dispensing rate	inhalers dispensed	CO_2e	dispensing rate	inhalers dispensed	CO_2e
Characteristic	(n/1,000)[3]	(n)	(t)	(n/1,000)[3]	(n)	(t)	(n/1,000)[3]	(n)	(t)	(n/1,000)[3]	(n)	(t)
ICS	6	175,914	209	30	768,543	13,773	12	334,039	449	34	891,842	16,142
ICS/LABA	109	3,174,565	2,722	94	2,703,912	71,552	113	3,343,878	2,846	106	3,057,584	83,251
ICS/LAMA/LABA	17	568,330	433	1	20,583	290	21	682,823	517	4	124,239	1,734
LABA	52	1,690,486	993	-	-	-	47	1,544,695	933	-	-	-
LAMA/LABA	21	672,001	475	-	-	-	20	656,091	468	-	-	-
SABA	1	43,899	22	172	4,892,399	137,388	3	109,005	54	186	5,363,856	150,613
SAMA	-	-	-	7	197,180	2,877	-	-	-	8	239,034	3,488
**Total**	207	6,325,195	4,854	303	8,582,617	225,880	215	6,670,531	5,266	338	9,676,555	255,227

[1]includes dry powder inhalers and soft mist inhalers

[2]includes pressurised metred dose inhalers and breath actuated inhalers

[3]age-standardised to Australian Standard Population (2010)[4]

MMPC= mean monthly percentage change, CI=confidence interval; CO_2e= carbon dioxide equivalent; ICS= inhaled corticosteroid; ICS/LABA= combined inhaled corticosteroid/ long-acting $beta_2$ agonist; ICS/LAMA/LABA= combined inhaled corticosteroid/ long-acting muscarinic antagonist/ long-acting $beta_2$ agonist; LABA= long-acting $beta_2$ agonist; LAMA/LABA= combined long-acting muscarinic antagonist / long-acting $beta_2$ agonist; n=number; SABA= short-acting $beta_2$ agonist; SAMA= short-acting muscarinic antagonist

Table 3c. Pharmaceutical Benefits Scheme-subsidised respiratory inhalers and estimated carbon emissions by demographic characteristics of users and inhaler class, 2023 and change 2019-2023

Year	2023[4]						Trend (2019-2023)			
	Low emissions inhalers[1]			High emissions inhalers[2]			Low emissions inhalers[1]		High emissions inhalers[2]	
	dispensing rate	inhalers dispensed	CO_2e	dispensing rate	inhalers dispensed	CO_2e	dispensing rate	CO_2e	dispensing rate	CO_2e
**Characteristic**	(n/1,000)[3]	(n)	(t)	(n/1,000)[3]	(n)	(t)	MMPC[5] (95% CI)		MMPC[5] (95% CI)	
**Area**										
Metropolitan	171	3,771,616	2,895	279	5,821,808	152,983	0.10% (-0.08% to 0.27%)	0.16% (-0.03% to 0.35%)	0.38% (0.11% to 0.66%)*	0.43% (0.16% to 0.72%)*
Non-metropolitan	282	2,518,092	1,917	422	3,419,819	89,093	0.27% (0.16% to 0.38%)*	0.29% (0.1% to 0.41%)*	0.48% (0.27% to 0.68%)*	0.53% (0.34% to 0.73%)*
**State/ Territory**										
Australian Capital Territory	211	101,880	80	245	114,642	2,938	0.28% (0.10% to 0.46%)*	0.61% (0.41% to 0.80%)*	0.19% (-0.09% to 0.46%)	0.42% (0.14% to 0.70%)*
New South Wales	200	2,026,437	1,550	313	2,945,634	76,950	-0.11% (-0.28% to 0.05%)	0.07% (-0.09% to 0.24%)	0.15% (-0.11% to 0.41%)	0.31% (0.04% to 0.58%)*
Northern Territory	133	27,903	21	222	49,419	1,263	-0.13% (-0.25% to -0.01%)*	0.33% (0.18% to 0.48%)*	0.33% (0.19% to 0.47%)*	0.56% (0.41% to 0.72%)*
Queensland	207	1,348,481	1,022	312	1,888,295	49,921	-0.00% (-0.17% to 0.17%)	0.27% (0.10% to 0.43%)*	0.28% (-0.02% to 0.59%)	0.51% (0.21% to 0.82%)*
South Australia	199	487,289	368	432	924,060	24,377	0.04% (-0.13% to 0.22%)	0.23% (0.05% to 0.40%)*	0.30% (0.02% to 0.59%)*	0.48% (0.18% to 0.77%)*
Tasmania	249	190,633	149	445	300,698	7,899	-0.00% (-0.15% to 0.14%)	0.23% (0.08% to 0.38%)*	0.39% (0.19% to 0.60%)*	0.57% (0.35% to 0.93%)*
Victoria	195	1,553,710	1,195	313	2,337,682	61,428	0.07% (-0.10% to 0.25%)	0.29% (0.11% to 0.48%)*	0.47% (0.19% to 0.76%)*	0.64% (0.35% to 0.93%)*
Western Australia	167	553,560	427	216	680,589	17,279	0.01% (-0.18% to 0.21%)	0.31% (0.11% to 0.50%)*	0.34% (-0.02% to 0.69%)	0.59% (0.24% to 0.95%)*
**Age group**										
0-19 years	16	102,973	84	179	1,142,721	28,677	0.40% (0.05% to 0.76%)*	0.49% (0.12% to 0.85%)*	0.12% (-0.34% to 0.58%)	0.18% (-0.28% to 0.65%)
20-39 years	65	483,678	399	136	1,021,717	27,906	0.17% (-0.05% to 0.39%)	0.16% (-0.07% to 0.38%)	0.25% (-0.09% to 0.60%)	0.37% (0.03% to 0.71%)*

Year	2023[4]						Trend (2019-2023)			
	Low emissions inhalers[1]			High emissions inhalers[2]			Low emissions inhalers[1]		High emissions inhalers[2]	
	dispensing rate	inhalers dispensed	CO_2e	dispensing rate	inhalers dispensed	CO_2e	dispensing rate	CO_2e	dispensing rate	CO_2e
**Characteristic**	(n/1,000)[3]	(n)	(t)	(n/1,000)[3]	(n)	(t)	MMPC[5] (95% CI)		MMPC[5] (95% CI)	
40-59 years	197	1,324,468	1,058	323	2,156,665	57,780	0.10% (-0.07% to 0.26%)	0.14% (-0.02% to 0.32%)	0.35% (0.10% to 0.61%)*	0.45% (0.20 to -0.71%)*
60-79 years	658	3,201,096	2,416	774	3,772,976	98,272	-0.12% (-0.30% to 0.07%)	0.20% (0.03% to0.38%)*	0.33% (0.03% to 0.64%)*	0.58% (0.28% to 0.87%)*
80 years and older	1,036	1,177,910	856	1,017	1,156,313	29,637	-0.05% (-0.23% to 0.13%)	0.33% (0.15% to0.50%)*	0.34% (0.03% to 0.66%)*	0.61% (0.30% to 0.91%)*
**Gender**										
Female	208	3,459,568	2,659	341	5,270,350	138,739	0.04% (-0.14% to 0.22%)	0.26% (0.08% to 0.43%)*	0.31% (0.03% to 0.59%)*	0.50% (0.23% to 0.78%)*
Male	185	2,830,679	2,154	277	3,971,897	103,352	-0.08% (-0.24% to 0.08%)	0.16% (0.00% to 0.32%)*	0.27% (0.02% to 0.52%)*	0.44% (0.19% to 0.69%)*
**Class**										
ICS	9	249,496	312	26	706,460	12,689	0.95% (0.30% to 1.60%)*	1.18% (0.45% to 1.90%)*	0.01% (-0.41% to 0.44%)	0.10% (-0.30% to 0.51%)
ICS/LABA	102	3,102,212	2,515	99	2,939,986	80,976	-0.04% (-0.24% to 0.16%)	-0.03% (-0.23% to0.16%)	0.46% (0.21% to 0.71%)*	0.80% (0.55% to 1.04%)*
ICS/LAMA/LABA	25	827,083	627	9	306,852	4,287	1.92% (1.73% to 2.11%)*	2.16% (1.95% to 2.36%)*	10.79% (9.20% to 12.48%)*	11.12% (9.59% to 12.71%)*
LABA	41	1,388,033	860	-	-	-	-0.80% (-0.95% to -0.65%)*	-0.27% (-0.41% to -0.12%)*	-	-
LAMA/LABA	18	627,473	451	-	-	-	-0.18% (-0.37% to 0.02%)	0.14% (0.00% to 0.28%)*	-	-
SABA	3	96,729	48	170	5,040,602	140,510	7.60% (4.02% to 11.28%)*	8.25% (4.44% to 12.19%)*	0.13% (-0.16% to 0.43%)	0.29% (0.00% to 0.57%)*
SAMA	-	-	-	8	252,611	3,686	-	-	0.95% (0.60% to 1.31%)*	1.14% (0.81% to 1.48%)*
**Total**	198	6,291,026	4,813	312	9,246,511	242,148				

[1]includes dry powder inhalers and soft mist inhalers

[2]includes pressurised metred dose inhalers and breath actuated inhalers

[3]age-standardised to Australian Standard Population (2010)[4]

[4]data only available for January to November. Totals and rates were multiplied by 1.099 to provide an estimate for the whole year

[5]MMPC= a summary measure of the trend between January 2019 and December 2023, calculated as a mean of all monthly percentage changes over the entire time period.* indicates that the MMPC is significantly different from zero

* indicates that the MMPC is significantly different from zero. For these estimates data for March 2020 was replaced by the mean from March 2019 and March 2021 to minimise impact of Covid-19 pandemic

MMPC= mean monthly percentage change, CI=confidence interval; CO_2e= carbon dioxide equivalent; ICS= inhaled corticosteroid; ICS/LABA= combined inhaled corticosteroid/ long-acting $beta_2$ agonist; ICS/LAMA/LABA= combined inhaled corticosteroid/ long-acting muscarinic antagonist/ long-acting $beta_2$ agonist; LABA= long-acting $beta_2$ agonist; LAMA/LABA= combined long-acting muscarinic antagonist / long-acting $beta_2$ agonist; n=number; SABA= short-acting $beta_2$ agonist; SAMA= short-acting muscarinic antagonist

Figure 1. Pharmaceutical Benefits Scheme-subsidised dispensing of respiratory inhalers and estimated carbon emissions, Australia, 2019–2023, by inhaler class and month[1]

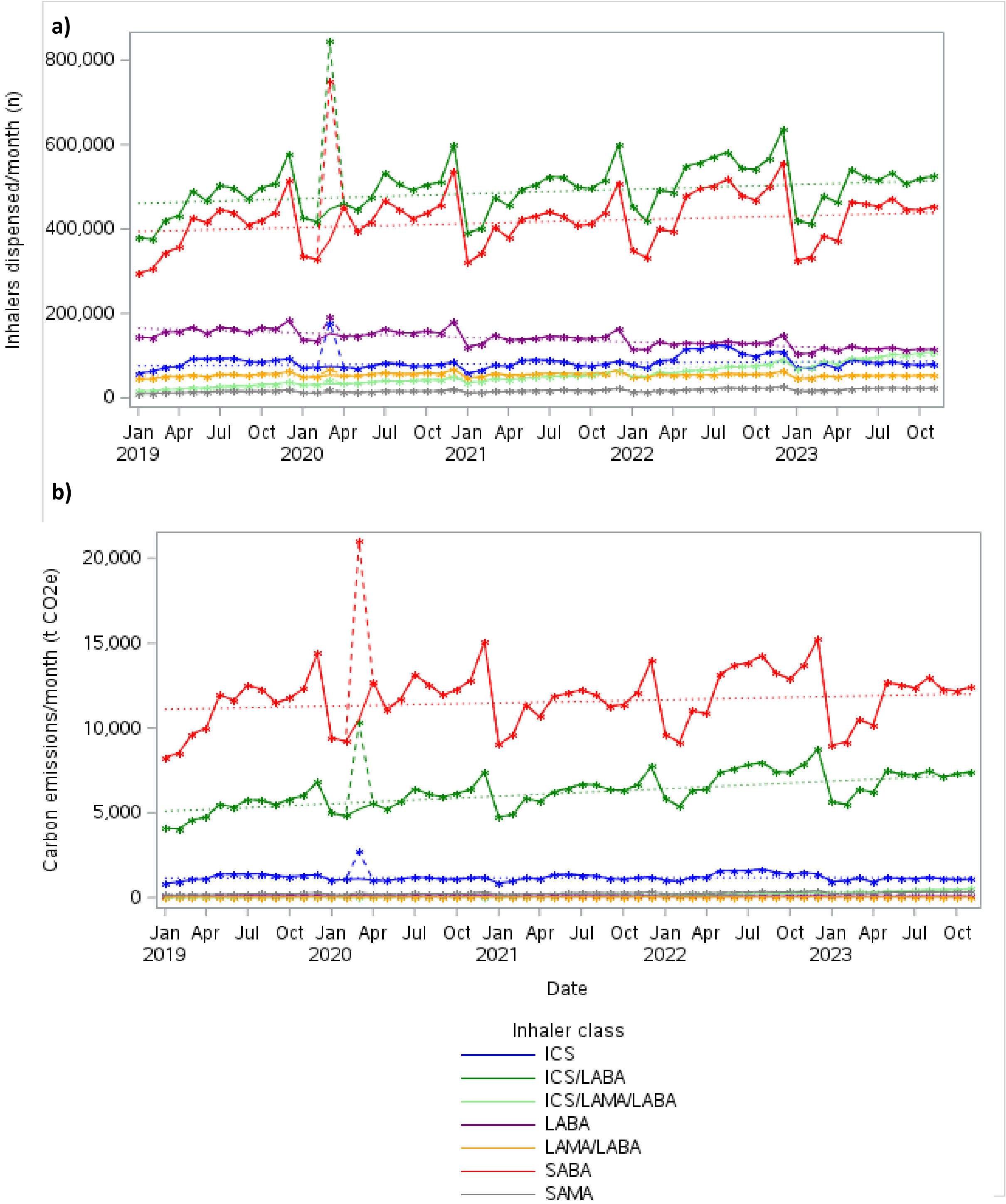

* Solid line indicates actual dispensing, dotted line indicates trend line, dashed line around March 2020 indicates outliers due to Covid-19 pandemic stockpiling

CO2e= carbon dioxide equivalent; ICS= inhaled corticosteroid; ICS/LABA= combined inhaled corticosteroid/ long-acting beta2 agonist; ICS/LAMA/LABA= combined inhaled corticosteroid/ long-acting muscarinic antagonist/ long-acting beta2 agonist; LABA= long-acting beta2 agonist; LAMA/LABA= combined long-acting muscarinic antagonist / long-acting beta2 agonist; n=number; SABA= short-acting beta2 agonist; SAMA= short-acting muscarinic antagonist
